# A Moss 2-Oxoglutarate/Fe(II)-Dependent Dioxygenases (2-ODD) Gene of Flavonoids Biosynthesis Positively Regulates Plants Abiotic Stress Tolerance

**DOI:** 10.3389/fpls.2022.850062

**Published:** 2022-07-29

**Authors:** Huijuan Wang, Shenghao Liu, Fenghua Fan, Qian Yu, Pengying Zhang

**Affiliations:** ^1^National Glycoengineering Research Center and School of Life Science, Shandong University, Qingdao, China; ^2^Key Laboratory of Marine Eco-Environmental Science and Technology, First Institute of Oceanography, Ministry of Natural Resources, Qingdao, China; ^3^Shandong Key Laboratory of Carbohydrate Chemistry and Glycobiology, Shandong University, Qingdao, China

**Keywords:** abiotic stress, Antarctic moss, anthocyanin accumulation, 2-oxoglutarate/Fe(II)-dependent dioxygenases (2-ODDs), flavonoids, flavonol

## Abstract

Flavonoids, the largest group of polyphenolic secondary metabolites present in all land plants, play essential roles in many biological processes and defense against abiotic stresses. In the flavonoid biosynthesis pathway, flavones synthase I (FNSI), flavanone 3-hydroxylase (F3H), flavonol synthase (FLS), and anthocyanidin synthase (ANS) all belong to 2-oxoglutarate/Fe(II)-dependent dioxygenases (2-ODDs) family, which catalyzes the critical oxidative reactions to form different flavonoid subgroups. Here, a novel *2-ODD* gene was cloned from Antarctic moss *Pohlia nutans* (*Pn2-ODD1*) and its functions were investigated both in two model plants, *Physcomitrella patens* and *Arabidopsis thaliana*. Heterologous expression of *Pn2-ODD1* increased the accumulation of anthocyanins and flavonol in *Arabidopsis*. Meanwhile, the transgenic *P. patens* and *Arabidopsis* with expressing *Pn2-ODD1* exhibited enhanced tolerance to salinity and drought stresses, with larger gametophyte sizes, better seed germination, and longer root growth. Heterologous expression of *Pn2-ODD1* in *Arabidopsis* also conferred the tolerance to UV-B radiation and oxidative stress by increasing antioxidant capacity. Therefore, we showed that *Pn2-ODD1* participated in the accumulation of anthocyanins and flavonol in transgenic plants, and regulated the tolerance to abiotic stresses in plants, contributing to the adaptation of *P. nutans* to the polar environment.

## Introduction

The 2-oxoglutarate/Fe(II)-dependent dioxygenases (2-ODDs) are non-heme iron-containing soluble proteins, which catalyze the oxidation reactions of diverse substrates, including hydroxylation, demethylation, desaturation, halogenation, and epimerization (Wang et al., 2021c; Wei et al., 2021). 2-ODDs mainly participate in the biosynthesis of various metabolic pathways, such as flavonoids, benzylisoquinoline alkaloids, glucosinolates, tropane alkaloids, and plant hormones (Kawai et al., 2014; Wang et al., 2019). Flavonoids are the largest class of specialized metabolites in plants, which have been isolated and identified more than 10,000 species. Despite their diversity, they all share the basic 15-carbon phenylpropanoid core (C6–C3–C6 skeleton structure) (Li C. et al., 2017). Flavonoids biosynthesis starts with the general phenylpropanoid pathway to produce *p*-coumaroyl-CoA. Then, *p*-coumaroyl-CoA and malonyl-CoA were catalyzed by chalcone synthase (CHS) and chalcone isomerase (CHI) to form naringenin, a key precursor of flavonoids biosynthesis (Jiang et al., 2016; Busche et al., 2021). Naringenin can be either converted by flavone synthase I and/or II (FNSI or FNSII) to produce flavone, or by flavanone-3-hydroxylase (F3H) to form dihydrokaempferol (DHK). Consequently, DHK can be converted to flavonols or anthocyanins by flavonol synthase (FLS) or dihydroflavonol reductase (DFR) and anthocyanin synthase (ANS) (Ahn et al., 2015; Wang et al., 2021b). Moreover, the modifications and derivatization of the carbon atoms of the basic skeleton, catalyzed by glycosyltransferase and O-methyltransferases, result in the diverse flavonoid groups in nature, including isoflavones, flavones, flavanones, flavanols, flavonols, and anthocyanins (Farrow and Facchini, 2014). Among the enzymes in the flavonoid biosynthesis pathway, flavones synthase I (FNSI), flavanone 3-hydroxylase (F3H), flavonol synthase (FLS), and anthocyanidin synthase (ANS)/leucoanthocyanidin dioxygenase (LDOX) belong to the 2-ODDs family, which requires 2-oxoglutarate, Fe^2+^, and ascorbate as co-factors to catalyze a series of oxidation reactions (Park et al., 2019; Busche et al., 2021).

FNSIs and F3Hs exhibit a relatively limited substrate selectivity, whereas FLSs and ANSs can accept a wide range of flavonoid compounds as possible substrates (Li et al., 2020a). FNSIs from primitive land plants (*Plagiochasma appendiculatum, Physcomitrella patens*, and *Selaginella moellendorffii*) displayed FNS and F3H dual activities, catalyzing naringenin into apigenin and DHK (Han et al., 2014; Li et al., 2020a). PcFNSI from *Petroselinum crispum* converted (2R,3S)-cis-DHK to kaempferol *in vitro* (Martens et al., 2003). F3Hs share high similarity with FNSIs and convert (2S)-flavanones into dihydroflavonols. FLSs are responsible for flavonol biosynthesis, in competition with DFR in the anthocyanin pathway for their common substrates DHK (Martens et al., 2010). Overexpression of FLSs could lead to the accumulation of flavonol and the reduction of anthocyanin in plant tissues (Luo et al., 2015; Jiang et al., 2020). *In vitro*, recombinant FLS protein from *Oryza sativa* not only converted DHK and dihydroquercetin (DHQ) into kaempferol and quercetin, but also catalyzed eriodictyol and naringenin to form DHQ and DHK, exhibiting FLS and F3H activities (Park et al., 2019). FLS from *Citrus unshiu* also catalyzed the unnatural (2R)-naringenin and natural (2S)-naringenin to yield dihydrokaempferol and kaempferol, respectively (Lukačin et al., 2003). FLSs share identity with ANSs at the polypeptide level and accept (2R,3S,4R)-leucoanthocyanidins as substrate (Turnbull et al., 2004). ANS can react with leucoanthocyanidins, flavanones, and dihydroflavonols, as well as (+)-Catechin, displaying a broad variety of substrate selectivity (Park et al., 2019). Functional defects or silencing of ANS affect the formation of plant color, thus resulting in the colorless or white organs (Rafique et al., 2016).

Flavonoids are extensively distributed in extant land plants, including bryophytes and vascular plants, which is thought to be crucial for plants land colonization and adaptation to terrestrial ecosystems (Jiang et al., 2016; Li et al., 2020a; Piatkowski et al., 2020). During plant evolution, different classes of flavonoids appeared sequentially (Koes et al., 1994). First, chalcones, flavanones, and flavones were found in the basal land plants liverworts due to the existence of functional FNSI, whereas proanthocyanidins were present in lycophytes (ferns). Furthermore, in addition to the generation of dominant flavone, both ferns and gymnosperms began to synthesize flavonols. In *P. patens*, flavonols were also detected (Wolf et al., 2010). Finally, flavonols and anthocyanins appeared with the emergence of angiosperms, harboring the true F3H genes (Li et al., 2020a). Flavonols participate in UV-B protection, male fertility, and regulating plant growth and development (Hamamouch et al., 2020). Anthocyanins, a water-soluble plant pigments, accountable for red to purple colors, are abundant in many plant tissues in seed plants (Tanaka et al., 2008; Pervaiz et al., 2017), which are involved in many critical biological functions, including attracting pollinators for pollination, providing resistance of herbivory, and absorbing UV-B radiation. (Ahn et al., 2015; Pervaiz et al., 2017). However, the downstream branch pathway of flavonoids biosynthesis in the early terrestrial plant bryophytes is not clear, and whether bryophytes can synthesize anthocyanins is still controversial.

Non-vascular plant liverworts can produce cell wall-localized red flavonoid pigment riccionidins (an auronidin), which is formed by a branch of phenylpropanoid metabolism pathway distinct from anthocyanins biosynthesis (Kunz et al., 1993; Berland et al., 2019). And moss *Sphagnum capillifolium* had been reported to generate sphagnorubins (Mues, 2000), described as “anthocyanin-like” pigments, which contributes to alleviating abiotic stresses, much like the anthocyanins in seed plants (Albert et al., 2018; Piatkowski et al., 2020). In *P. patens*, several probable 2-oxoglutarate-dependent dioxygenase genes were from its genome, and 2-ODD1 from *P. patens* exhibited FNSI/F3H activity and catalyzed naringenin to produce apigenin and dihydrokaempferol (Li et al., 2020a). However, there were no detectable anthocyanin pigments in stressed *P. patens* by high-performance thin-layer chromatography (Wolf et al., 2010). A phylogenetic analysis revealed that investigated seedless plants' liverworts, mosses, and ferns possessed no orthologs of ANS, and orthologs representing the complete anthocyanin biosynthetic pathway only existed in the seed plants (Piatkowski et al., 2020). However, some 2-ODD genes from *S. moellendorffii* and *P. patens* were also annotated as probable ANS. Furthermore, six anthocyanin compounds including peonidin 3-O-glucoside chloride, peonidin O-hexoside, pelargonidin, malvidin 3-O-glucoside, cyanidin 3-O-galactoside, and cyanidin 3-O-rutinoside were recently confirmed to be present in Antarctic moss *Leptobryum pyriforme* by a widely targeted metabolomics, and cyanidin 3-O-rutinoside was the significantly up-regulated metabolite with log2(Fold change) 14.68 under ultraviolet-B radiation (Liu et al., 2021).

In Antarctica, extreme aridity, cold, and high UV-B radiation severely restrict the growth of terrestrial plants (Singh et al., 2010). The Antarctic plants have evolved independently for millions of years due to the separation of Antarctica from other continents. Mosses, one of the dominant terrestrial plants in the limited ice-free ground, have evolved a series of special physiological mechanisms to adapt tough environments (Convey et al., 2014; Alavilli et al., 2017). For example, in three East Antarctic mosses, the accumulation of cell wall UV-B-absorbing compounds and red pigments acted as a photoprotective mechanism against UV-B radiation (Waterman et al., 2018). Antarctic moss *Andreaea regularis* displayed high levels of carotenoids and UV-B screening pigments in foliage in response to UV-B exposure (Newsham, 2003). Bioflavonoids extracted from Antarctic mosses *Ceratodon purpureus* exhibited UV screening and antioxidant activity, which may be accountable for the high resistance to UV-B radiation (Waterman et al., 2017). The transcriptome profiling of Antarctic moss *Pohlia nutans* under UV-B treatment indicated that the antioxidant system, DNA-repairing system, and flavonoids biosynthesis pathway contributed to the adaptation and survival of moss to UV-B radiation (Li et al., 2019). Antarctic moss *Sanionia uncinata* exhibited tolerance to desiccation due to the activation of protective mechanisms that are involved in increased activity of antioxidant enzymes, accumulation of osmotic adjustment compounds like proline, glycine betaine, and dehydrins proteins (Pizarro et al., 2019). In this study, we isolated a 2-ODD1 gene from the Antarctic moss *P. nutans* (*Pn2-ODD1*) and investigated its functions in *P. patens* and *Arabidopsis*. These results showed that heterologous expression of *Pn2-ODD1* promoted the accumulation of anthocyanins and flavonol in transgenic plants, and conferred plant tolerance to salt, drought, and UV-B stress by increasing antioxidant capacity.

## Materials and Methods

### Plant Materials and Growth Conditions

*Arabidopsis thaliana* (Col-0) was used as the WT plant and for the generation of transgenic lines. *Arabidopsis* was grown in a greenhouse at 22°C and 60% relative humidity, with 8-h light/16-h dark (light intensity, 100 μmol·m^−2^·s^−1^) for vegetative growth. After about 4 weeks, they were cultured in a growth chamber with 16-h light for subsequent reproductive maturation. *Physcomitrella patens* were cultivated on BCD solid medium at 25°C, in 16-h light/8-h dark photoperiod (60 μmol·m^−2^·s^−1^). For the production of protonema cells, *P. patens* were homogenized by a polytron homogenizer and cultivated on agar medium (BCD, supplemented with 5 mM diammonium tartrate) overlaid cellophane for convenient subculture.

### Bioinformatics Analysis

The sequence of *Pn2-ODD1* gene was obtained from the transcriptome of Antarctic moss *Pohlia nutans* using HMMER program (Li et al., 2019). Subsequently, the multiple sequence alignments of Pn2-ODD1 and other plant 2-ODDs were performed using DNAMAN software. The neighbor-joining method was used to construct the phylogenetic tree using MEGA 5.0 software.

### RNA Isolation and Quantitative Real-Time PCR Analysis

Total RNA was extracted from *Arabidopsis* and *P. patens* by TRIzol reagent and CTAB method. cDNA was synthesized from 2 μg RNA by 5 × All-In-One RT MasterMix (abm, Canada), according to the manufacturer's instructions. Then, quantitative real-time PCR analysis was performed in an LC480 Thermal Cycler instrument by using the SYBR qPCR Master Mix (Nuoweizan, Nanjing, China). *AtTubulin* and *PpActin* were used as an internal control. The expression levels of genes were presented by the comparative 2^−Δ*ΔCt*^ method (Livak and Schmittgen, 2001). All used primers were listed in Supplementary Table 1.

### Plasmid Constructions and Plant Transformation

The full length of *Pn2-ODD1* was amplified by PCR from cDNA of *Pohlia nutans* with the specific primers. And the PCR fragment was inserted into the *SmaI* restriction site of the pTFH15.3 vector by one-step cloning (Nuoweizan, Nanjing, China). Then, the constructed *Pn2-ODD1*-pTFH15.3 plasmid was linearized by *NotI* to introduce into *P. patens* protoplasts by PEG-mediated DNA uptake, with a slight modification (Cove et al., 2009). Different concentrations of antibiotic G418 (25, 50, or 100 μg·mL^−1^) were used to select surviving transformants. Finally, stable transformants were screened by PCR amplification for phenotypic analysis.

For expressed-*Pn2-ODD1 Arabidopsis, Pn2-ODD1* gene was cloned into the pRI101 vector to obtain *Pn2-ODD1*-pRI101. The recombinant construct was then introduced into *Arabidopsis* (Col-0) plants *via Agrobacterium*-mediated infiltration method (Zhang et al., 2006). Positive transgenic seedlings were screened on 1/2 MS medium supplemented with 50 μg·mL^−1^ kanamycin. Two independent T3 *Pn2-ODD1* transgenic lines were obtained for subsequent analysis.

### Measurement of Anthocyanin and Flavonols Contents

For the observation of anthocyanin enrichment in 5-day-old *Arabidopsis* seedlings, the seeds were cultivated on 1/2 MS agar medium, with 24-h light for 5 days at 22°C (Li P. et al., 2017). For the accumulation of anthocyanins in 17-day-old seedlings, sterilized seeds were germinated on 1/2 MS solid medium containing 3% (w/v) sucrose in a culture room with 16-h light for 14 days, then transferred on 1/2 MS medium supplemented with 12% (w/v) sucrose for another 3 days (Yonekura-Sakakibara et al., 2012). The purple coloration of seedlings was recorded, and plant samples were collected for the spectrophotometry measurement of anthocyanin levels and the analysis of flavonoids or anthocyanins metabolomics.

Anthocyanin extraction from *Arabidopsis* was carried out as described in Xu et al. (2017). Briefly, homogenized samples were incubated with methanol-HCl (1%, v/v) at 4°C for 24 h in the dark. Then, the extracts were mixed with chloroform and distilled water to remove chlorophyll. After centrifugation at 12,000 g for 15 min, the supernatant was collected and measured the absorbance at 657 and 530 nm. The anthocyanin level was expressed as (A530-0.25^*^A657)/fresh weight (g).

The total flavonols were extracted from *Arabidopsis* according to the method of Wang et al. (2020), with a slight modification. 0.2 g of fresh samples were ground with liquid nitrogen and extracted using 50% methanol. After centrifugation at 13,000 g for 15 min, the supernatant was transferred into a new tube, followed by adding an equal volume of 2 N HCl and hydrolyzing at 70°C for 40 min. Then, 100% methanol was added to prevent the degradation of the aglycones. The mixture was centrifuged for 15 min at 13,000 g to collect the supernatants for HPLC analysis (Shimadzu LC-20A, Japan), equipped with an Agilent C18 column (4.6 ×250 mm, 5 μm). The mobile phase was 0.1% (v/v) formic acid (A) and 0.1% (v/v) formic acid in acetonitrile (B). The linear gradient conditions were as follows: 0 min, 5% B; 30 min, 55% B; 45 min, 65% B; 50 min, 100% B; 52-60 min, 5% B, at a flow rate of 1 mL·min^−1^. The column temperature was set at 30°C, and the flavonols were detected at 365 nm (Park et al., 2019).

### Targeted Metabolomics Analysis

The flavonoids and anthocyanins metabolomics analyses in *Arabidopsis* plants were performed by Wuhan Metware Biotechnology Co., Ltd. (Wuhan, China). In general, the freeze-dried sample was homogenized into powder. For widely targeted flavonoids metabolomics, 0.1 g powder was dissolved with 70% aqueous methanol at 4°C overnight. After centrifugation, the filtered supernatant was analyzed using LC-MS/MS (UPLC, Shim-pack UFLC SHIMADZU CBM30A system; MS, Applied Biosystems 6500 Q TRAP). Based on a self-built database and a public metabolite database, the metabolites were qualitatively identified by secondary spectral properties. Differential metabolites with VIP (variable importance in the project) ≥1, fold change ≥1.2, or fold change ≤ 0.83 were filtered as significantly changed metabolites.

For targeted anthocyanins metabolomics analysis, 50 mg homogenized powder was incubated with an extraction solvent containing 0.5 mL methanol/water/hydrochloric acid (500:500:1, V/V/V) by vortex and ultrasound for 5 min. After that, the sample was centrifuged at 12,000 g at 4°C for 3 min. Finally, the supernatants were collected and filtrated to analyze by Ultra Performance Liquid Chromatography and ESItriple quadrupole-linear ion trap mass spectrometer (QTRAP). For the absolute quantitative analysis of anthocyanins, commercial standard substances were diluted to different concentrations to obtain the corresponding mass spectrum peak intensity. Then, the absolute contents of anthocyanins in extracted samples were calculated by the standard curves of different substances. The differential metabolites were selected according to the *P* values (≤ 0.05) and fold change (≥1.2 or ≤ 0.83).

### Plant Stress Treatments

For stress treatments of transgenic *P. paten*, the stem tips with the same size of transgenic *Pn2-ODD*1 and WT gametophytes were placed on a BCD medium supplemented with different concentrations of NaCl and D-Mannitol for 5 weeks at 25°C. The visual phenotypes were photographed, and the colony diameters were measured.

For drought stress assay, 3-week-old *Arabidopsis* seedlings grown in soil containers were subjected to drought stress by depriving water for 21 days, and then rewatered for 3 days to calculate the survival rates. For the seed germination of *Arabidopsis*, the sterilized seeds were sown on the 1/2 MS solid medium with or without NaCl, d-mannitol, H_2_O_2_, and 3-amino-1,2,4-triazole (3-AT, a CAT inhibitor, triggering the accumulation of H_2_O_2_). Then plates were placed in the dark at 4°C for 2 days and then cultured in a greenhouse at 22°C for 4–8 days. The germination rate was represented by counting the proportion of cotyledon greening. For root length assay, seedlings were vertically grown on a 1/2 MS medium containing 75 or 100 mM NaCl, 0.2 or 0.3 M d-mannitol, and 0.75 or 1 mM H_2_O_2_. After 5–9 days, the root length was calculated using Image J software. For the expression analysis of genes under stress treatment, 2-week-old *Arabidopsis* were sprayed with 200 mM NaCl, 16% PEG, or 20 mM H_2_O_2_ for 2 h, and all plant samples were harvested and frozen at −80°C until use.

For UV-B treatment, 3-week-old WT and transgenic *Pn2-ODD1 Arabidopsis* seedlings were treated for 12 h with 0.25 mW·cm^−2^ UV-B intensity. After being recovered for 3 days, the seedlings were collected and immediately frozen in liquid nitrogen and stored at −80°C for further analysis. *Arabidopsis* cultivated under the normal light conditions was used as the control.

### Chlorophyll Content Quantification

For the measurement of chlorophyll content, homogenized samples were extracted with 80% acetone at 4°C in the dark. After centrifugation, the supernatant was collected to detect the absorbance at 663 and 645 nm by spectrophotometry analysis. Total chlorophyll content was calculated using the following formula: (8.05 × A663 + 20.29 × A645) × mL acetone mg^−1^ fresh weight (Porra et al., 1989).

### 3,3′-Diaminobenzidine (DAB) and Nitrobluetetrazolium Blue Chloride (NBT) Staining

DAB and NBT staining were used to detect the accumulation of hydrogen peroxide (H_2_O_2_) and superoxide anion (${\text{O}}_{2}^{-}$) as described previously (Wang et al., 2015), with a slight modification. Whole seedlings were incubated with 1 mg·mL^−1^ DAB solution or 1 mg·mL^−1^ NBT solution in 50 mM potassium phosphate buffer (pH7.8) for 12 h in the dark. Then, chlorophyll was completely removed by decolorization with a bleaching solution (ethanol:acetic acid:glycerol = 3:1:1) in a boiling water bath.

### Statistical Analysis

All experiments were performed at least three times for biological replicates. All data were expressed as mean ± standard deviation (SD).

## Results

### Characterization and Sequence Analysis of *Pn2-ODD1* Gene

**Figure 1:**
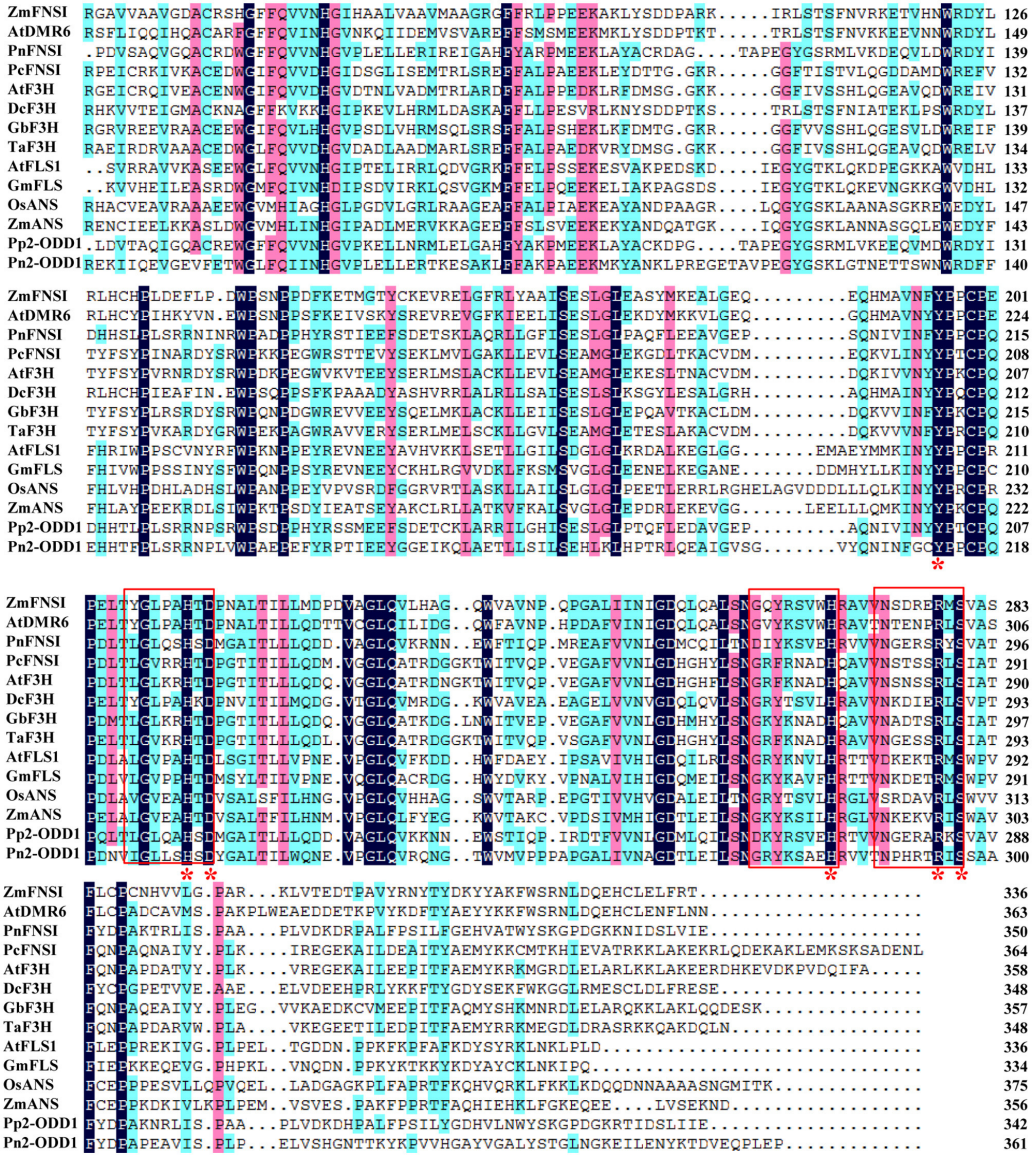
Pn2-ODD1 shared homology with other known species 2-ODDs. The amino acid sequence alignment of Pn2-ODD1 with 2-ODDs protein from other species. Red frames indicated ferrous iron binding site HXDX55H and 2-oxoglutarate (2-ODD) binding site RXS. Red asterisks (*) indicated the conserved amino acid residues of 2OG-FeII_Oxy conserved domain. *Zea mays*, ONL99521.1_ZmFNSI1; *Arabidopsis thaliana*, Q9FLV0.1_AtDMR6; *Pohlia nutans*, QCP71067.1_PnFNSI; *Petroselinum crispum*, AAP57393.1_PcFNSI; *Arabidopsis thaliana*, NP_190692.1_AtF3H; *Dendrobium catenatum*, PKU66857.1_DcF3H; *Ginkgo biloba*, AAU93347.1_GbF3H; *Triticum aestivum*, AFJ38181.1_TaF3H; *Arabidopsis thaliana*, NP_001190266.1_AtFLS1; *Glycine max*, NP_001237419.1_GmFLS; *Oryza sativa*, CAA69252.1_OsANS; *Zea mays*, and NP_001106074.1_ZmANS; *Physcomitrella patens*, XP_001780809.1_Pp2-ODD1.

**Figure 2:**
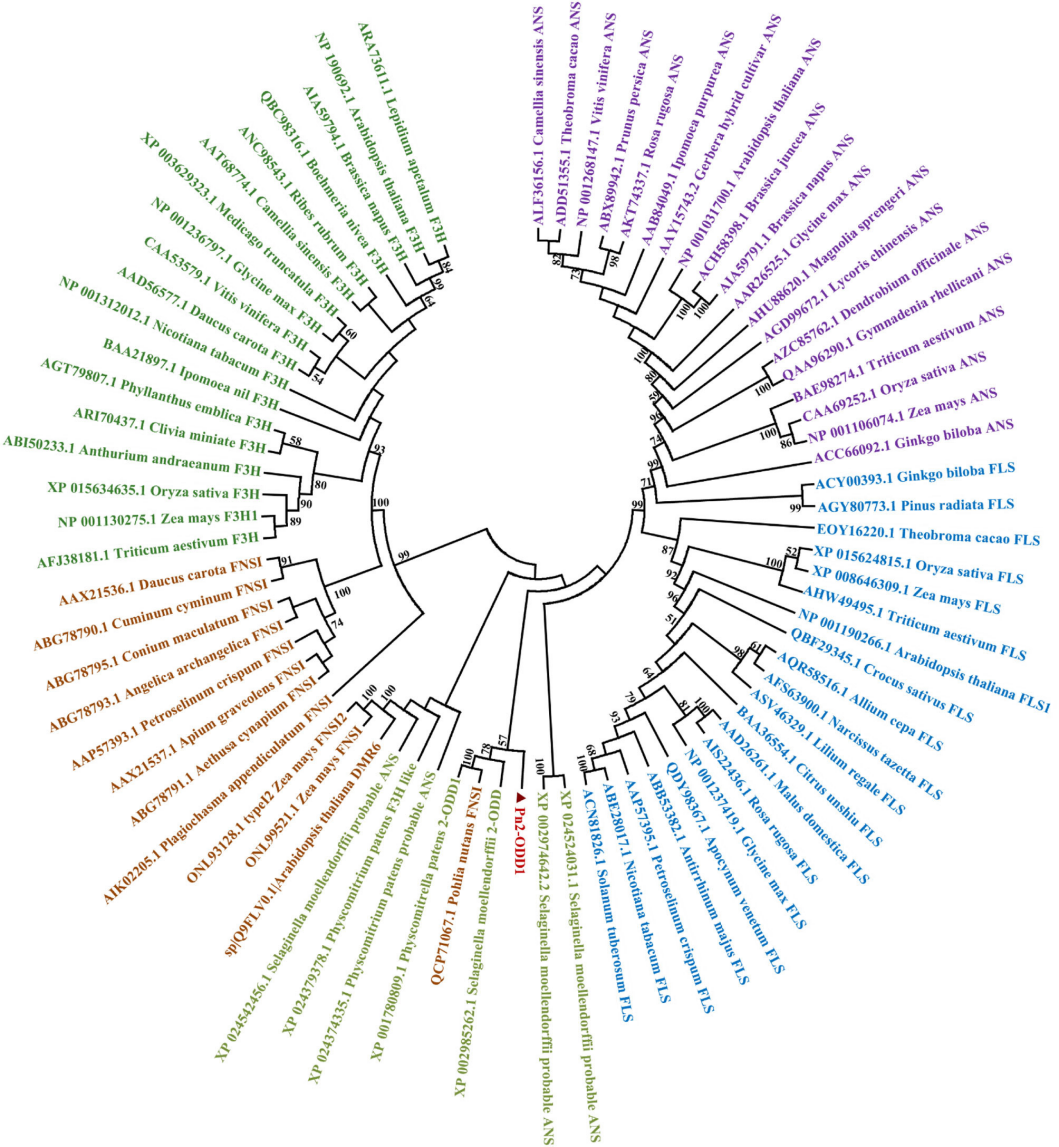
Phylogenetic relationship of Pn2-ODD1 and other known representative 2-ODDs (mosses *Physcomitrella patens* and *Pohlia nutans*, ferns *Selaginella moellendorffii*, gymnosperms, monocotyledons, and dicotyledons).

The full-length cDNA sequence of *Pn2-ODD1* was amplified by PCR, which contains an open reading frame of 1,086 bp, encoding a 40.3 kDa-polypeptide with 361 amino acids and a predicted isoelectric point of 5.53. Multiple alignment analysis showed that Pn2-ODD1 shared only 35.3% similarity with the 2-ODD1 from *P. paten* and 36.7% similarity with FNSI from *P. nutans*, whereas about 25.0% identity with other known species 2-ODDs. However, Pn2-ODD1 contained the conserved domains: Fe^2+^ binding site HxDxnH (His^228^, Asp^230^, and His^285^) and the 2-oxoglutarate (2-OG) binding domain RxS (Tyr^213^, Arg^295^, and Ser^297^) (Figure 1). Phylogenetic analysis showed that in the members of a 2-ODDs family in flavonoids biosynthesis pathway, ANSs, and FLSs belonged to one subclade, while Apiaceae FNSIs and F3Hs constituted another independent branch. Pn2-ODD1 clustered with FLSs and ANSs branch and was closely related to *S. moellendorffii* and *P. patens* 2-ODDs proteins (Figure 2).

### *Pn2-ODD1* Contributed to the Accumulation of Anthocyanin and Flavonols in Transgenic *Arabidopsis*

**Figure 3:**
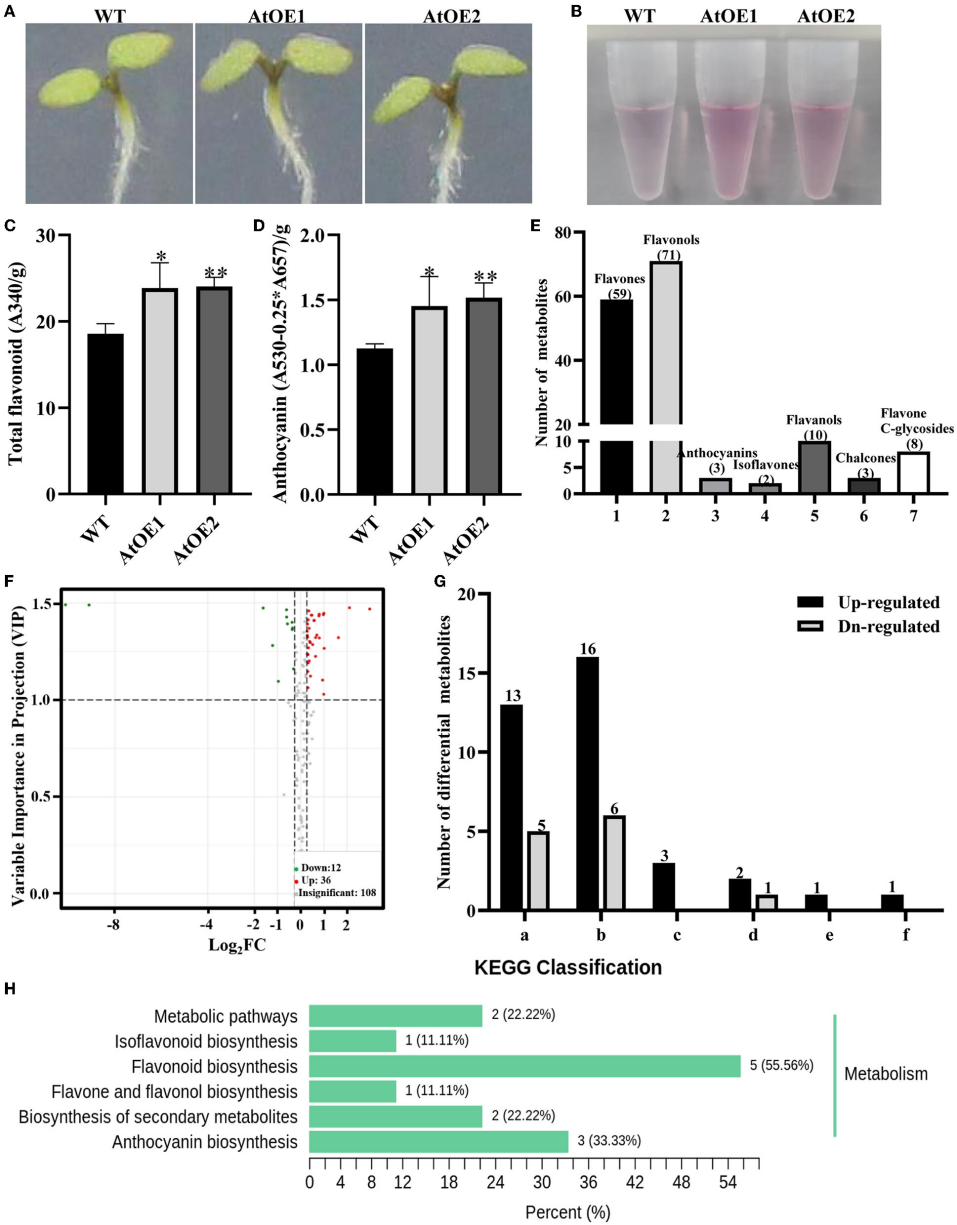
*Pn2-ODD1* increased the levels of flavonoids in 5-day-old *Arabidopsis*. **(A)** Anthocyanin accumulation visualized by the purple coloration in 5-day-old seedlings with constant light. **(B)** Anthocyanin extraction solutions for WT and AtOE lines. **(C,D)** Contents of total flavonoids and anthocyanins in 5-day-old seedlings detected by spectrophotometry analysis. **(E)** Number of different classes of flavonoids in 5-day-old WT and transgenic *Arabidopsis* detected by the flavonoids metabolomics. **(F)** Volcano plot of samples. The volcano plot showed the levels of flavonoids metabolites and the statistical significance. Each point represents a metabolite. Horizontal ordinate indicates the fold change of flavonoids metabolites between WT and OE1, while VIP value means a significant difference in statistical analysis. **(G)** Statistical analysis of the classes of differential flavonoid metabolites. **(a–f)** Flavones, flavonols, anthocyanins, flavanols, chalcones, and flavone C-glycosides. **(H)** Differential metabolites KEGG classification. Asterisk (*) represents a significant difference between the WT plants and AtOE lines (Student's t-test, *P < 0.05, **P < 0.01).

*Arabidopsis* is a model organism in the plant kingdom, due to the easily quantitative stress-resistance indicators, clear flavonoids biosynthesis pathway, and regulatory networks, which is beneficial to promote the substantial development and application of genetic resources in higher plants. To explore the biological function of *Pn2-ODD1*, two independently expressed *Pn2-ODD1 Arabidopsis* were identified by genomic PCR (Supplementary Figure 1). Constant light can induce the accumulation of flavonoids in plants. After the sterilized seeds were sown on the 1/2 MS medium and placed vertically with the constant light for 5 days, both WT and *Pn2-ODD1*-expressed *Arabidopsis* displayed the accumulation of flavonoids, with the purple hypocotyls. However, AtOE lines accumulated higher total flavonoids and anthocyanin levels (spectrophotometry analysis), with the deeper hypocotyls, which were 1.30- and 1.35-fold in contrast with WT plants, respectively (Figures 3A–D). The flavonoids metabolomics of 5-day-old WT and AtOE lines were detected based on UPLC-MS/MS method. A total of 156 metabolites were identified, including 59 flavones, 71 flavonols, two isoflavones, 10 flavanols, three chalcones, three anthocyanins, and eight flavone C-glycosides (Figure 3E). According to VIP value ≥ 1 and fold-change threshold ≥ 1.2 or ≤ 0.83, 48 differential metabolites (36 up-regulate and 12 down-regulate) were screened (Figure 3F and Supplementary Table 2). Among them, flavonols were the most abundant compounds (16 up-regulated and six down-regulated) (Figure 3G). Notably, three detected anthocyanins were all up-regulated. KEGG classification analysis showed that the differential metabolites dominantly focused on the flavonoid and anthocyanin biosynthesis metabolism (Figure 3H).

**Figure 4:**
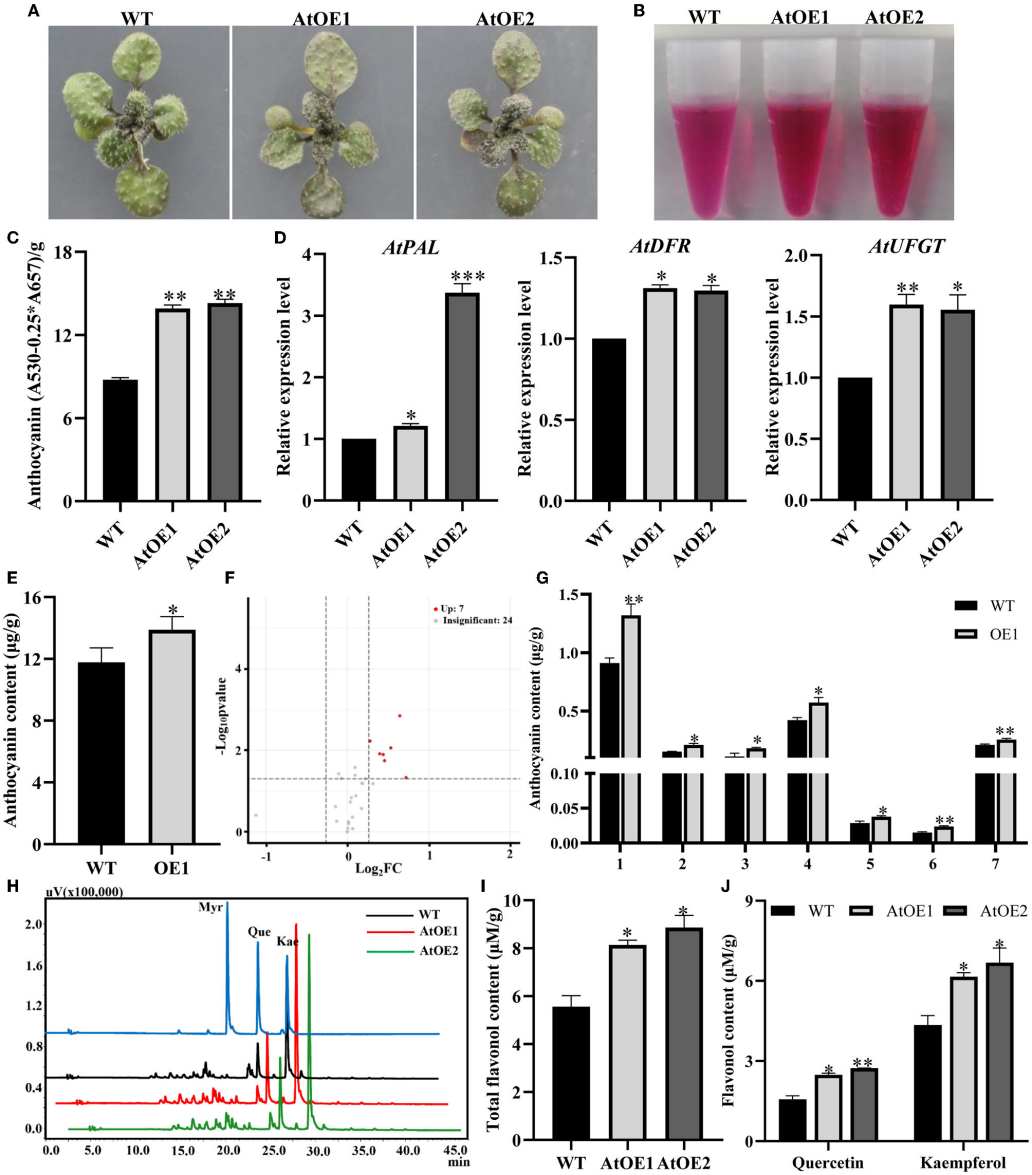
*Pn2-ODD1* enhanced the accumulation of anthocyanins and flavonols in 17-day-old *Arabidopsis* seedlings induced by sucrose. **(A)** Anthocyanin accumulation visualized by the purple coloration in 17-day-old seedlings induced by sucrose (cultured on a medium containing 3% sucrose for 14 days, then transferred to 12% sucrose medium for 3 days). **(B,C)** Anthocyanin extraction solutions and anthocyanins contents (spectrophotometry analysis). **(D)** Expression levels of genes in anthocyanin biosynthesis pathway in WT and AtOE lines induced by sucrose. **(E)** The absolute quantitative analyses of total anthocyanins in 17-day-old *Arabidopsis* seedlings induced by sucrose detected by targeted metabolomics strategy. **(F)** Volcano plot of samples. It showed the levels of anthocyanins metabolites and the statistical significance of the difference. Each point represents a metabolite. Horizontal ordinate represents the fold change of anthocyanins metabolites between two samples, while *p*-value means significant difference in statistical analysis. **(G)** 7 up-regulated anthocyanins metabolites identified in the transgenic *Pn2-ODD1 Arabidopsis*. 1–7: Cyanidin-3,5-O-diglucoside, cyanidin-3-O-rutinoside, cyanidin-3-O-(6-O-malonyl-beta-D-glucoside), cyanidin-3-O-5-O-(6-O-coumaryl)-diglucoside, delphinidin-3-O-rutinoside, peonidin-3,5-O-diglucoside, and peonidin-3-O-(6-O-p-coumaryl)-glucoside. **(H)** HPLC profiles of flavonols standards (myricetin, quercetin, and kaempferol) and the flavonols extracts from WT and expressed-*Pn2-ODD1 Arabidopsis* (365 nm). **(I,J)** Contents of total flavonol, quercetin, and kaempferol in WT and AtOE lines induced by sucrose. Asterisk (*) represents a significant difference between the WT plants and AtOE lines (Student's *t*-test, *P < 0.05, **P < 0.01, ***P < 0.001).

Also, when cultivated on 1/2 MS medium containing sucrose, transgenic *Pn2-ODD1 Arabidopsis* exhibited greater anthocyanin accumulation, with deeper purple leaves, which was about 1.60-fold in contrast with WT plants (Figures 4A–C). Meanwhile, the expression levels of anthocyanin synthesis pathway genes *AtPAL, AtDFR*, and *AtUFGT* were increased in AtOE lines (Figure 4D). To further determine the difference in anthocyanin metabolism profiles, we performed the targeted anthocyanin metabolomics of AtOE lines and WT plants induced by sucrose. As shown in Figure 4E, the OE1 line had a 17.6% increase in the levels of total anthocyanins than that of the WT plants, which was consistent with the result measured by spectrophotometry analysis (anthocyanins at A530 nm). Totally, 31 anthocyanin metabolites were detected, including cyanidin (14), delphinidin (5), petunidin (3), pelargonidin (3), and peonidin (6). Based on a fold-change threshold ≥ 1.2 or ≤ 0.83, 7 up-regulated differential metabolites were identified (Figure 4F). Among them, cyanidin-3-O-(6-O-malonyl-beta-d-glucoside) and peonidin-3,5-O-diglucoside were the most significantly differential metabolites with log2(fold change), which were 1.65 and 1.56 folds compared with WT plants, respectively (Figure 4G). Then, the content of flavonol (including quercetin and kaempferol) in the WT plants and expressed-*Pn2-ODD1 Arabidopsis* grown with sucrose was analyzed using HPLC. The results showed that there was no significant difference in flavonol components (mainly quercetin and kaempferol) between WT plants and AtOE lines. However, the content of total flavonols in transgenic *Arabidopsis* was increased by 50.0%, in contrast with WT plants (Figures 4H,I). Among them, the levels of quercetin and kaempferol were significantly enhanced, which were about 65.0% and 50.0% higher than that of the WT plants, respectively (Figure 4J). These results suggested that the heterologous expression of *Pn2-ODD1* resulted in the enrichment of anthocyanin and flavonols in *Arabidopsis*.

### *Pn2-ODD1* Conferred the Tolerance to Salt Stress in Transgenic *Physcomitrella patens* and *Arabidopsis*

**Figure 5:**
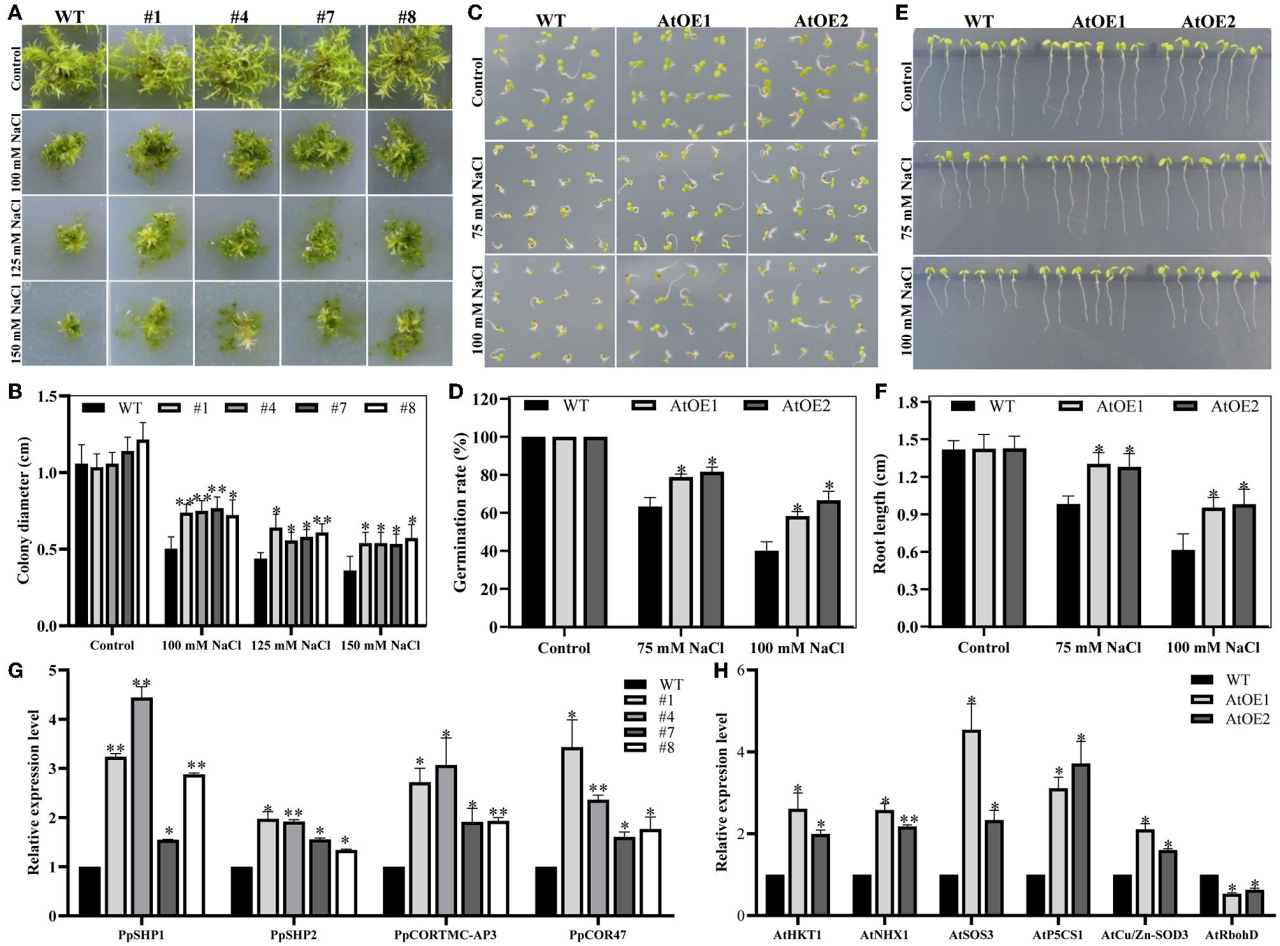
Heterologous expression of *Pn2-ODD1* enhanced plant resistance to salt stress. **(A)** The transgenic *P. patens* displayed larger gametophytes than the WT plants under NaCl treatment. **(B)** Statistical analysis of gametophyte size as shown in **(A)**. **(C,E)** AtOE lines exhibited higher germination rates and longer root length in contrast with WT plants under salt stress. **(D,F)** Statistical analysis of the seed germination rates and root length as shown in **(C,E)**. **(G,H)** Expression patterns of stress-responsive genes in *Pn2-ODD1* transgenic *P. patens* and *Arabidopsis* measured by qRT-PCR. Asterisk (*) represents a significant difference between the WT plants and AtOE lines (Student's *t*-test, *P < 0.05, **P < 0.01).

*P. patens* has become an important model plant for functional gene research due to its rapid growth cycle and mature genetic transformation methods (He et al., 2019; Rensing et al., 2020). Four independent transgenic *P. patens* (#1, #4, #7 and #8) were obtained and confirmed by genomic PCR analysis to further determine the function of *Pn2-ODD1* in response to abiotic stress (Supplementary Figure 1). The stem tips with the same size of transgenic lines and WT plants were grown on BCD solid medium containing 100, 125, and 150 mM NaCl. Under normal medium, there was no obvious difference in growth performance between expressed-*Pn2-ODD1 P. patens* and WT plants. However, in the presence of NaCl, the transgenic *P. patens* displayed larger gametophytes than the WT plants. On 100 mM NaCl medium, the clone size of WT plants was 5.03 mm, whereas those of transgenic *P. patens* were 7.39, 7.50, 7.67, and 7.22 mm. On 125 or 150 mM NaCl, the diameter of the gametophyte of transgenic *P. patens* was still larger than that of the wild type, which was about an increase of 1.35- and 1.50-fold, respectively (Figures 5A,B). In *Arabidopsis*, no detectable differences in germination rate and root length were observed between WT and AtOE lines in the absence of NaCl. On 1/2 MS mediums supplemented with 75 mM NaCl, the germination rate of expressed-*Pn2-ODD1* plants was 78.0% and 81.7%, which was about 15.0% higher than that of the WT plants. Similarly, at 100 mM NaCl, AtOE lines also displayed better germination, with an increase of 20.0% in comparison with WT plants (Figures 5C,D). For root length assays, the early root length in the transgenic *Pn2-ODD1 Arabidopsis* was markedly more vigorous, which was about 1.30-fold or 1.55-fold longer than that of the WT plants at 75 mM NaCl or 100 mM NaCl (Figures 5E,F).

To further investigate the possible molecular mechanism of increased tolerance to salt stress in transgenic *Pn2-ODD1* lines, the transcript levels of stress-responsive genes were analyzed by qRT-PCR. We found that the expression patterns of salt tolerance genes *PpSHP1* and *PpSHP2*, stress responsible genes *PpCOR TMC-AP3* and *PpCOR47* were markedly up-regulated in transgenic *P. patens*, in contrast with WT plants (Figure 5G). Meanwhile, the expression patterns of salt tolerance genes *AtHKT1, AtNHX1, AtSOS3*, and *AtP5CS1*, and antioxidant enzymes gene *AtCu/Zn-SOD3* were markedly increased in AtOE lines, while ROS generation gene *AtRbohD* was down-regulated in expressed-*Pn2-ODD1 Arabidopsis* (Figure 5H). Therefore, these results suggested that enhanced salt tolerance by *Pn2-ODD1* might be correlated with upregulating several stress-related genes.

### *Pn2-ODD1* Enhanced the Resistance to Drought Stress in Transgenic Plants

**Figure 6:**
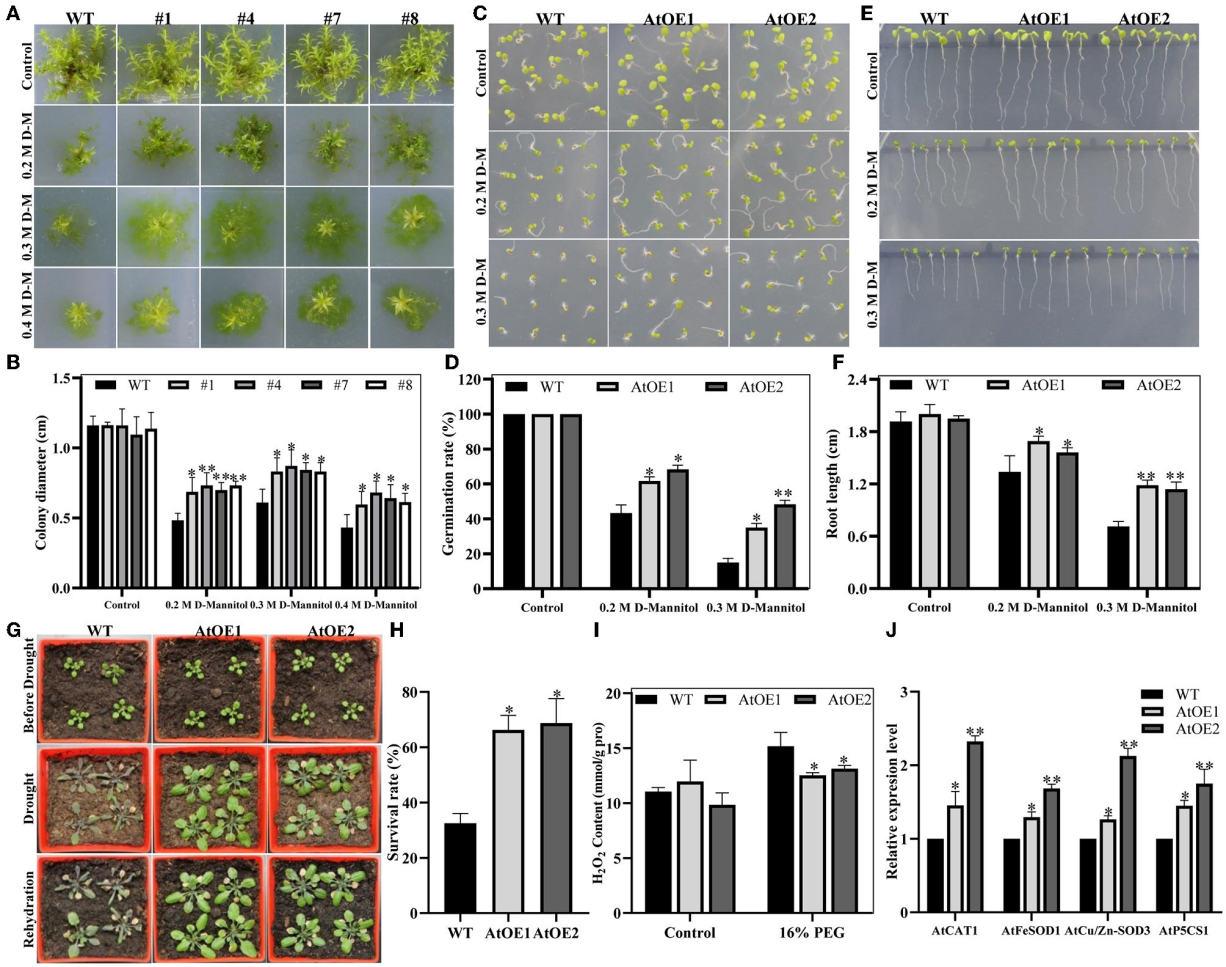
*Pn2-ODD1* increased the resistance to drought stress in transgenic *P. patens* and *Arabidopsis*. **(A)** The diameter of transgenic *P. patens* gametophytes was markedly larger than that of the WT plants under drought stress conditions. **(B)** Statistical analysis of gametophyte size as shown in **(A)**. **(C,E)** Expressed-*Pn2-ODD1 Arabidopsis* exhibited higher germination rates and longer root length compared with WT plants under osmosis stress. **(D,F)** Statistical analysis of the seed germination rates and root length as shown in **(C,E)**. **(G)** Three-week-old *Arabidopsis* seedlings grown in soil were withheld water for 21 days, and then rewatered for 3 days. **(H)** Statistical analysis of the survival rate, as shown in **G**. **(I)** H_2_O_2_ content in *Arabidopsis* under 16% PEG treatment. **(J)** Gene expression levels of ROS-scavenging related genes in *Arabidopsis* analyzed by qRT-PCR. Asterisk (*) indicates a significant difference between the WT plants and AtOE lines (Student's *t*-test, *P < 0.05, **P < 0.01).

Salt tolerance is usually associated with osmotic resistance. Thus, the growth performance of WT and expressed*-Pn2-ODD1* plants under drought stress was analyzed. As shown in Figures 6A,B, the heterologous expression of *Pn2-ODD1* significantly increased the resistance to d-mannitol stress in *P. patens*. On 0.3 M d-mannitol medium, the clone size of expressed-*Pn2-ODD1 P. patens* was 8.32, 8.73, 8.44, and 8.31 mm, which was about 40.0% higher than that of WT plants (6.11 mm), following the formation of protonema. When exposed to 0.4 M d-mannitol, the diameter of the protonema of transgenic *P. patens* was about 6.33 mm, which was 1.50-fold in contrast with WT plants. In *Arabidopsis*, 3-week-old seedlings with the same growth were treated with water withdrawal for 21 days. As shown in Figure 6G, AtOE lines exhibited better performance with a 65.0% survival rate, whereas WT plants were severely damaged and became wilted, with a 32.5% survival rate (Figure 6H). The sterilized seeds were sown on 1/2 MS medium supplemented with different concentrations of d-mannitol to observe the germination and root growth. On 0.2 M d-mannitol treatment, transgenic *Pn2-ODD1* lines displayed 61.6–68.3% germination rates, which was about 20.0% higher than WT plants (Figures 6C,D). The root resistance assays demonstrated that AtOE lines exhibited longer root length, which was about 1.20-fold at 0.2 M d-mannitol and 1.60-fold at 0.3 M d-mannitol, in contrast with WT plants (Figures 6E,F). Drought stress can result in the generation of reactive oxygen species (ROS). The contents of H_2_O_2_ in AtOE lines were significantly lower than those of WT plants after 16% PEG treatment (Figure 6I). Antioxidant enzymes, such as CAT and SOD, participate in the reduction process of ROS (Choudhury et al., 2013). qRT-PCR analysis showed that the expression levels of ROS-scavenging related gene *AtCAT1, AtFeSOD1, AtCu/Zn-SOD3*, and proline biosynthesis-related gene *AtP5CS1* were markedly increased in transgenic *Pn2-ODD1 Arabidopsis* (Figure 6J). These results demonstrated that *Pn2-ODD1* contributed to enhanced resistance to drought stress by increasing ROS clearance capacity in transgenic plants.

### Heterologous Expression of *Pn2-ODD1* Increased *Arabidopsis* Tolerance to UV-B Radiation and Oxidative Stress

**Figure 7:**
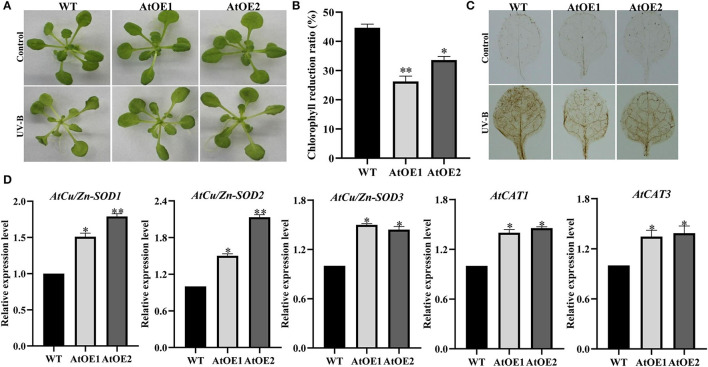
Heterologous expression of *Pn2-ODD1* enhanced the *Arabidopsis* resistance to UV-B radiation. **(A)** Three-week-old *Arabidopsis* were treated with 0.25 mW·cm^−2^ UV-B intensity for 12 h and then recovered for 3 days. **(B)** Chlorophyll degradation ratio of WT plants and AtOE lines. **(C)** Levels of hydrogen peroxide (H_2_O_2_) measured by 3,3′-diaminobenzidine (DAB) staining under UV-B radiation. **(D)** Gene expression patterns of antioxidant enzymes under UV-B treatment. Asterisk (*) represents a significant difference between the WT plants and AtOE lines (Student's *t*-test, *P < 0.05, **P < 0.01).

To investigate whether *Pn2-ODD1* could enhance the plant tolerance to UV-B stress, 3-week-old *Arabidopsis* were subjected to UV-B radiation with 0.25 mW·cm^−2^ UV-B intensity for 12 h and recovered for 3 days. When compared with WT plants, AtOE lines displayed better growth performance, with greener leaves and less damage (Figure 7A). The chlorophyll degradation ratio of transgenic *Pn2-ODD1 Arabidopsis* was lower than that of the WT plants under UV-B treatment (Figure 7B). DAB staining suggested that AtOE lines accumulated less endogenous H_2_O_2_ levels, in contrast with WT plants (Figure 7C). Furthermore, the transcript patterns of ROS-scavenging genes *AtCu/Zn-SOD1, AtCu/Zn-SOD2, AtCu/Zn-SOD3, AtCAT1*, and *AtCAT3* were up-regulated in expressed-*Pn2-ODD1 Arabidopsis* (Figure 7D).

**Figure 8:**
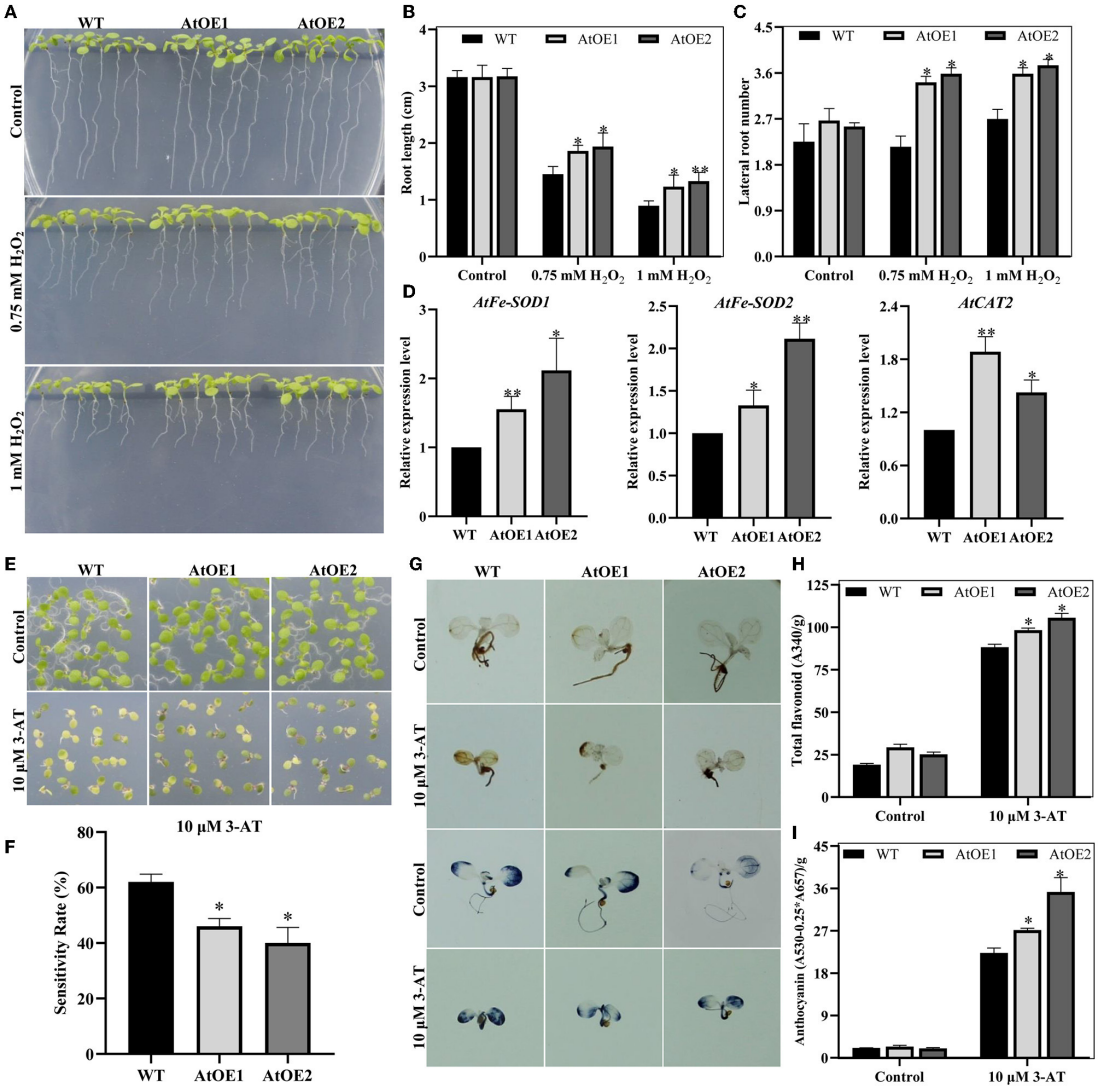
*Pn2-ODD1* conferred the tolerance to oxidative stress in *Arabidopsis*. **(A)** Primary root length of *Arabidopsis* seedlings under oxidative stress. **(B,C)** Statistical analysis of root length and lateral root number as shown in **(A)**. **(D)** Expression patterns of ROS-scavenging protein (i.e., *AtFeSOD1, AtFeSOD2*, and *AtCAT1*) in *Arabidopsis* under oxidative stress. **(E,F)** Growth phenotype and statistical analysis of WT and AtOE lines under the treatment of 10 μM 3-AT for 7 days. **(G)** 3,3′-diaminobenzidine (DAB) and nitrobluetetrazolium blue chloride (NBT) staining. **(H,I)** Content of total flavonoids and anthocyanins in WT and AtOE lines under 3-AT treatment for 7 days. Asterisk (*) represents a significant difference between the WT plants and AtOE lines (Student's *t*-test, *P < 0.05, **P < 0.01).

In general, abiotic stresses lead to a build-up of ROS, which results in oxidative stress (Cruz de Carvalho, 2008; He et al., 2018). H_2_O_2_ and 3-amino-1,2,4-triazole (3-AT, a CAT inhibitor, triggering the accumulation of H_2_O_2_) were used to simulate exogenous and endogenous oxidative stress for young seedlings, respectively. The results showed that heterologous expression of *Pn2-ODD1* conferred the enhanced tolerance to exogenous and endogenous oxidative stress in *Arabidopsis*. In the presence of 0.75 mM H_2_O_2_, the root length of AtOE lines was 1.86 and 1.94 cm, which was an increase of 30.5%, compared with the WT plants (Figures 8A,B). Furthermore, the lateral root numbers of AtOE lines were markedly higher than that of the WT plants, which were about 1.60-fold longer at 0.75 mM H_2_O_2_, whereas 1.35-fold longer at 1.0 mM H_2_O_2_ (Figure 8C). qRT-PCR analysis demonstrated that the expression levels of At*FeSOD1, AtFeSOD2*, and *AtCAT1*, encoding ROS-scavenging enzymes, were significantly up-regulated in expressed-*Pn2-ODD1 Arabidopsis* (Figure 8D). Under endogenous oxidative stress, AtOE lines displayed reduced sensitivity to 3-AT, with greener leaves and lower H_2_O_2_ and ${\text{O}}_{2}^{-}$ levels, in contrast with WT plants (Figures 8E–G). Previous reports had demonstrated that ROS can trigger the accumulation of anthocyanins (Xu et al., 2017). Consistent with the results, under the treatment of 3-AT for 7 days, the levels of total flavonoid and anthocyanins in AtOE lines were markedly increased by 15.0% and 40.0%, in contrast with WT plants, respectively (Figures 8H,I). Thus, these results illustrated that *Pn2-ODD1* improved the resistance to oxidative stress by increasing ROS scavenger and anthocyanins accumulation.

## Discussion

2-Oxoglutarate/Fe(II)-dependent dioxygenases (2-ODDs), non-heme iron-containing soluble proteins, participate in the synthesis of important plant hormones and diverse secondary metabolites (Farrow and Facchini, 2014; Wei et al., 2021). Flavonoids, the natural products present in plants, possess enormous chemical diversity due to the organization and modifications of the three-ring structure by 2-ODDs, cytochrome P450-dependent oxygenases (CYPs), glycosyltransferase, and O-methyltransferases (Wang et al., 2019). In flavonoids biosynthesis, the key enzymes of downstream branch pathways FNSI, F3H, FLS, and ANS all belong to the 2-ODDs family, which is involved in the synthesis of key flavonoids compounds, such as flavones, flavonols, and anthocyanins. During the plant land colonization, the acquisition of flavonoids is considered to be an important adaptation for defense against the adverse environmental stresses faced with the transition to a non-aquatic lifestyle (Albert et al., 2018). Bryophytes are thought to be the oldest terrestrial plants, which exhibit the simple morphological organization, but the complex chemical diversification (Wellman et al., 2003; Asakawa et al., 2013; Ludwiczuk and Asakawa, 2019). Although there are several reports on the function of enzymes (4CL, CHS, CHI, and FNSI) in the upstream flavonoids pathway in bryophytes (Han et al., 2014; Gao et al., 2015; Yu et al., 2015; Cheng et al., 2018), the enzymes responsible for the downstream branch pathways of flavanone in bryophytes remain unclear. In particular, whether anthocyanin and anthocyanin synthase are present in bryophytes is still controversial. In this study, a *Pn2-ODD1* gene from Antarctic moss *Pohlia nutans* and its roles in flavonoids metabolism and abiotic stresses were studied. The amino acid sequence of Pn2-ODD1 shared only 35.3% identity with 2-ODD1 of *P. patens* and 36.8% identity with PnFNSI, whereas below 30.0% sequence identity with 2-ODDs from other plants. But Pn2-ODD1 possessed the conserved Fe^2+^ binding domains and 2-oxoglutarate (2-OG) binding domains (Figure 1), which was consistent with other members of the 2OG-FeII_Oxy dioxygenase family (Reddy et al., 2007; Han et al., 2014; Zhang et al., 2016; Wang et al., 2020). Phylogenetic analysis demonstrated that Pn2-ODD1 had a closer relationship with ANSs and FLSs, which clustered with the 2-ODDs from *P. patens* and *S. moellendorffii* (Figure 2).

2-ODDs family of flavonoids synthesis pathway participate in catalyzing the oxidation of the flavonoid “C ring” to form different flavonoid subclasses. F3Hs and FNSIs can both react with flavanones, while FNSIs in primitive land plants also displayed F3H activity (Li et al., 2020a). Both FLSs and ANSs can accept the unnatural (2R)-naringenin and the natural (2S)-naringenin, dihydroflavonols as well as leucoanthocyanidins as substrates (Turnbull et al., 2000, 2004; Lukačin et al., 2003). ANS also catalyzes (+)-Catechin to cyanidin and a dimeric flavan-3-one (Wellmann et al., 2006). Although 2-ODDs displayed the versatility and mutual substitutability in substrate selectivity, they performed their respective major functions of the flavonoid biosynthesis metabolism in plants. The accumulation of flavonoids can be changed by operating the heterologous or homologous expression of genes in the flavonoid biosynthetic pathway to further clarify the function of 2-ODDs in plants. Overexpression of *MnFNSI* from *Morus notabilis* in tobacco increased the levels of flavones in leaves and decreased the accumulation of anthocyanin in flowers (Li et al., 2020b). Overexpression of *F3Ha* and *F3Hb* from *Camellia sinensis* significantly enhanced the accumulation of oligomeric proanthocyanidins and flavonol glycosides, whereas the contents of monocatechin derivatives were decreased in *Arabidopsis* (Han et al., 2017). Antisense down-regulation of ANS in *Medicago truncatula* caused a reduction of anthocyanins in foliar (Pang et al., 2007). The *ans/fls1-2* seedlings complemented *ANS* from *Musa* spp. exhibited significantly increased levels of anthocyanins, compared with *ans/fls1-2* plants (Busche et al., 2021). In this study, the contents of total flavonoids and anthocyanins in AtOE lines grown for 5 days were significantly increased in contrast with WT plants (Figures 3A–D). Metabolomics is a valuable approach to analyze the chemical complexity and measure the bioaccumulated phytochemicals, which reveals the relationship between the metabolites and the physiological state in response to environmental or genetic changes (Abdelhafez et al., 2020). The widely targeted flavonoids metabolomics analysis showed that although these significantly changed metabolites were mainly flavones and flavonols, three detected anthocyanins were found to be all significantly up-regulated (Figures 3E–H). At present, it is still controversial whether anthocyanin is synthesized in bryophytes and anthocyanidin synthase in lower plants has not been reported. Therefore, we focused on the effect of *Pn2-ODD1* on anthocyanin synthesis. Sucrose can induce the accumulation of anthocyanins by increasing the expression of anthocyanins biosynthesis genes in plants (Teng et al., 2005; Solfanelli et al., 2006; Yoon et al., 2021). When induced by sucrose, the spectrophotometry analysis and targeted anthocyanin metabolomics demonstrated that heterologous expression of *Pn2-ODD1* significantly enhanced the levels of anthocyanins (Figures 4A–G). Meanwhile, heterologous expression of *Pn2-ODD1* also promoted the accumulation of flavonols in *Arabidopsis* (Figures 4H–J).

Heterologous expression of *FLS* from *Muscari aucheri* in tobacco increased the total flavonol level, whereas the total anthocyanin content in the petals was reduced (Liu et al., 2019). The antisense expression of FLS genes led to a reduction in the contents of flavonols and an increase in the accumulation of anthocyanins, when compared with the non-transformed plants (Holton et al., 1993; Nielsen et al., 2002). Also, overexpression of *ANS* from *Theobroma cacao* in tobacco led to flower petal color changes, which was consistent with an increased level of anthocyanins in flower petals (Liu et al., 2013). Previously, transgenic rice with ANS accumulated the increased anthocyanins and flavonols, and the reduced proanthocyanin levels, suggesting that rice ANS may be a multifunctional dioxygenase (Reddy et al., 2007). Overexpressed-*RtLDOX2 Arabidopsis* exhibited the enhanced anthocyanin and flavonol contents, possibly due to the versatility and mutual substitutability of RtLDOX2 in anthocyanin and flavonol biosynthesis (Li et al., 2021). In *Arabidopsis, fls1-2* mutant seedlings displayed the decreased flavonol glycosides levels and accumulated the glycosylated forms of dihydrofavonols, and the FLS-like side activity of LDOX in plants resulted in the remaining flavonol glycoside accumulation (Stracke et al., 2009). Therefore, we concluded that *Pn2-ODD1* contributed to the accumulation of anthocyanins and flavonol in *Arabidopsis*.

2-ODDs in the flavonoids biosynthesis pathway are involved in the growth and development, as well as stress responses of plants (Wei et al., 2021). Salinity stress can adversely hamper plant germination and growth, transpiration, and photosynthesis, which is one of the major threats to plant productivity (Wang et al., 2021a). The accumulation of flavonoids can be induced in response to salt stress (Arif et al., 2020). Overexpressed-*CsF3H* (from *Camellia sinensis*) tobacco exhibited increased tolerance to salinity stress by improved antioxidant system (Mahajan and Yadav, 2014). Overexpression of *FLS1* from *Triticum aestivum* enhanced the root length of *Arabidopsis* seedlings under salinity stress (Wang et al., 2014a). Also, overexpression of *ANS* from *Morus alba* L. promoted the resistance to NaCl and mannitol stress in transgenic tobacco (Li et al., 2018). Similar resistant phenotypes were also observed in transgenic *Pn2-ODD1* plants. Heterologous expression of *Pn2-ODD1* enhanced the resistance to salinity stress in *P. patens* and *Arabidopsis*, with larger diameters of gametophytes, increasing seed germination and root elongation, when exposed to NaCl stress (Figure 5). Plant can regulate ion homeostasis and compartmentalization by ion influx in response to salinity stress (Gupta and Huang, 2014). PpSHP1 and PpSHP2, encoding a small hydrophobic protein with two transmembrane domains, are involved in the maintenance of membrane structure and function under environmental stress and preventing over-accumulation of K^+^ and Na^+^ ions (Wang et al., 2014b). PpCOR47, the homolog of *Arabidopsis* LEA-like protein, protects cells from water stress and can be induced by salt and osmotic stresses (Gilmour et al., 1992). PpCOR TMC-AP3, homologous to chloroplastic amino acid-selective channel protein from barley, regulates the amino acid transportation between chloroplast and cytoplasm to resynthesize damaged proteins (Frank et al., 2005). In this study, the expression patterns of *PpSHP1, PpSHP2, PpCOR47*, and *PpCOR TMC-AP3* were significantly increased in transgenic *Pn2-ODD1 P. patens* under NaCl stress (Figure 5G). HKT and NHX, encoding K^+^ transporters and Na^+^/H^+^ exchangers, participate in maintaining ion homeostasis by controlling the transportation of Na^+^ and K^+^ during salinity stress (Gupta and Huang, 2014). SOS3 encodes a myristoylated Ca^2+^ binding protein with three EF hands, which is involved in salt tolerance through mediating Ca^2+^-dependent microfilament (MF) reorganization (M. Ishitani et al., 2000). P5CS1 catalyzes Glu to form Glu semialdehyde and is the key enzyme in the biosynthesis of proline, which is an osmoprotectant and stabilizes cellular structures, and keeps redox equilibrium in abiotic stresses (Aleksza et al., 2017). Overexpression of *AvFLS* from *Apocynum venetum* enhanced salt stress tolerance in tobacco by maintaining Na^+^/K^+^ homeostasis and increasing antioxidant enzyme activity (Wang et al., 2021a). We found that the transcript levels of *AtHKT1, AtNHX1, AtSOS3*, and *AtP5CS1* were obviously up-regulated in transgenic *Pn2-ODD1 Arabidopsis* (Figure 5H).

Drought stress can lead to a decline in quantity and quality of crop production (Naing et al., 2018). The biosynthesis of flavonoids in plants can be up-regulated when subjected to drought stress (Ma et al., 2014). Metabolome and transcriptome profiling in *Arabidopsis* showed that the overaccumulation of flavonoids was involved in enhanced tolerance to drought and oxidative stress in MYB overexpressors, transparent testa4 (*tt4*), and WT plants (Nakabayashi et al., 2014). Overexpression of *PnFNSI* contributed to the increased drought resistance by enhancing the antioxidant capacity in *Arabidopsis* (Wang et al., 2020). Overexpression of *RtLDOX2* from *Reaumuria trigyna* conferred enhanced tolerance to drought, UV-B, and salt stress in *Arabidopsis* by promoting the levels of anthocyanins and flavonols (Li et al., 2021). Here, heterologous expression of *Pn2-ODD1* contributed to the improved tolerance to drought stress in plants (Figure 6). The generation of ROS can be caused by drought stress. Reducing ROS enrichment in transgenic plants usually increased the resistance to drought stress (Choudhury et al., 2013). Transgenic tobacco overexpressing *F3H* from *Lycium chinense* displayed enhanced resistance to drought stress, improving the antioxidant system (Song et al., 2016). In this study, after 16% PEG treatment, *Pn2-ODD1* decreased the levels of H_2_O_2_ in *Arabidopsis* (Figure 6I). Superoxide dismutase (SOD) and catalase (CAT) are important antioxidant enzymes, which participate in the process of scavenging ROS in plants. SOD catalyzes the conversion of superoxide anions ${\text{O}}_{2}^{-}$ into H_2_O_2_ and O_2_, and CAT converts H_2_O_2_ to produce H_2_O and O_2_ (Sharma et al., 2012). qRT-PCR analysis showed that *Pn2-ODD1* markedly up-regulated the expression patterns of *AtCAT1, AtFeSOD1*, and *AtCu/Zn-SOD3* under 16% PEG treatment (Figure 6J).

Due to lower stratospheric ozone levels, plants are exposed to increased solar UV-B irradiation, which leads to oxidative damage to DNA, proteins, and lipids (Wolf et al., 2010). Flavonoids compounds, as a non-enzymatic antioxidant system, exhibit high antioxidant activity to protect plants from UV-B and oxidative damage (Buer et al., 2010). In *Arabidopsis*, anthocyanin-deficient mutants generated more ROS *in vivo*, accompanied by reduced antioxidant ability (Xu et al., 2017). Overexpression of rice ANS in rice mutant Nootripathu (NP) exhibited enhanced antioxidant activity by promoting the accumulation of anthocyanin and other flavonoids (Reddy et al., 2007). Overexpressing *ZmFNSI* or *ZmFNSII Arabidopsis* displayed less UV-B-induced damage due to the accumulation of apigenin, when compared with WT plants (Righini et al., 2019). In our study, heterologous expression of *Pn2-ODD1* conferred the tolerance to UV-B stress, exhibiting lower ROS levels and increased expression of antioxidant enzymes genes in *Arabidopsis* (Figure 7). Consistent with these results, we observed that heterologous expression of *Pn2-ODD1* in *Arabidopsis* conferred the tolerance to exogenous oxidative stress with longer primary roots and up-regulated the transcript levels of ROS scavenging gene, *AtFeSOD1, AtFeSOD2*, and *AtCAT1* (Figures 8A–D). ROS can induce the production of anthocyanins, and anthocyanin-deficient mutants accumulated more endogenous ROS and were hypersensitive to ROS (Xu et al., 2017). 3-AT, a CAT inhibitor, was used to stimulate endogenous oxidative stress. AtOE lines seedlings displayed lower sensitivity to ROS, less ROS accumulation, and markedly increased total flavonoids and anthocyanin levels, when exposed to 3-AT treatment (Figures 8E–I).

In conclusion, our results provided some evidence that a *Pn2-ODD1* gene from *P. nutans* increased the accumulation of anthocyanin and flavonol in transgenic plants. Also, heterologous expression of *Pn2-ODD1* conferred the plant resistance to salinity, drought, and UV-B stress, which may play a key role in the adaptation of *P. nutans* to the polar environment.

## Data Availability Statement

The original contributions presented in the study are included in the article/Supplementary Material, further inquiries can be directed to the corresponding author.

## Author Contributions

PZ and SL designed and supervised the experiments. HW conducted the experiments and wrote the manuscript. FF and QY assisted in phenotype analysis. HW and PZ analyzed and discussed the results. All authors agreed to publish the manuscript.

## Funding

This work was supported by the National Natural Science Foundation of China (41976225), Key Technology Research and Development Program of Shandong Province (2019GSF107064), Scientific Fund for National Public Research Institutes of China (GY0219Q05), and Central Government Guide Local Science and Technology Development Funds (YDZX20203700002579).

## Conflict of Interest

The authors declare that the research was conducted in the absence of any commercial or financial relationships that could be construed as a potential conflict of interest.

## Publisher's Note

All claims expressed in this article are solely those of the authors and do not necessarily represent those of their affiliated organizations, or those of the publisher, the editors and the reviewers. Any product that may be evaluated in this article, or claim that may be made by its manufacturer, is not guaranteed or endorsed by the publisher.

## Supplementary Material

Supplementary Figure 1 Gene transformation process of *Physcomitrella patens* and *Arabidopsis*. **(A)** Undigested *P. patens* protonema. **(B, C)** Digested *P. patens* protoplasts. **(D, E)** Screening of transgenic *Pn2-ODD1 P. patens* and *Arabidopsis* by antibiotic. **(F, G)** Identification of *Pn2-ODD1* in *P. patens* and *Arabidopsis* by PCR analysis.

Supplementary Table 1 All primers used for vector construction and gene expression analysis.

Supplementary Table 2 Differential metabolites (36 up-regulate and 12 down-regulate) identified by the flavonoids metabolomics in 5-day-old WT and transgenic *Arabidopsis*.
