# Supplementary figures and images for: A Moss 2-Oxoglutarate/Fe(II)-Dependent Dioxygenases (2-ODD) Gene of Flavonoids Biosynthesis Positively Regulates Plants Abiotic Stress Tolerance
Source: Front Plant Sci. 2022 Jul 29;13:850062. doi: 10.3389/fpls.2022.850062 (PMC9372559; doi:10.3389/fpls.2022.850062)

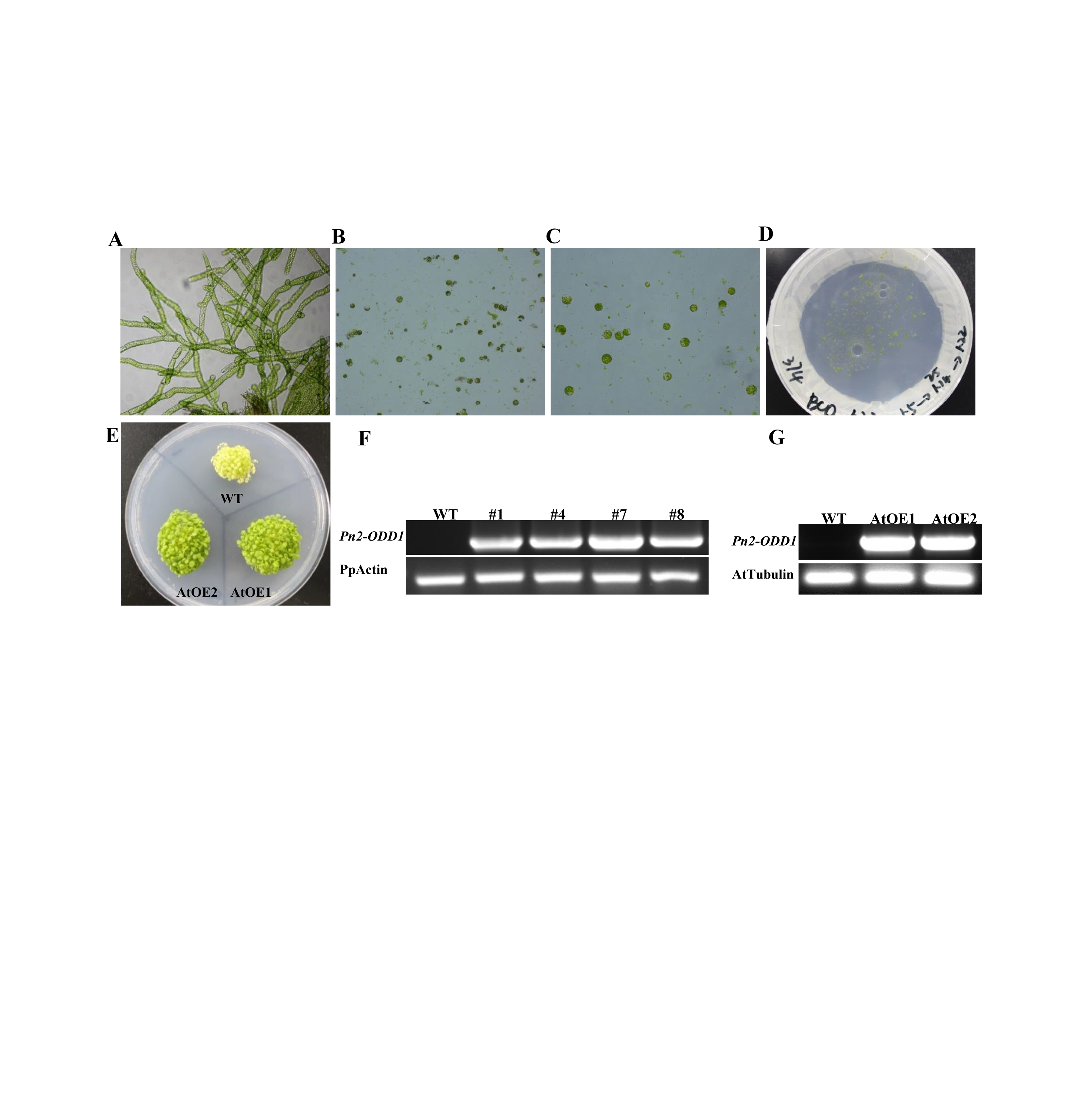

Supplement: Supplementary Figure 1 — Gene transformation process of Physcomitrella patens and Arabidopsis. (A) Undigested P. patens protonema. (B, C) Digested P. patens protoplasts. (D, E) Screening of transgenic Pn2-ODD1 P. patens and Arabidopsis by antibiotic. (F, G) Identification of Pn2-ODD1 in P. patens and Arabidopsis by PCR analysis. [file Image_1.tif]
