# Supplementary material for: A Moss 2-Oxoglutarate/Fe(II)-Dependent Dioxygenases (2-ODD) Gene of Flavonoids Biosynthesis Positively Regulates Plants Abiotic Stress Tolerance
Source: Front Plant Sci. 2022 Jul 29;13:850062. doi: 10.3389/fpls.2022.850062 (PMC9372559; doi:10.3389/fpls.2022.850062)
Supplement: Supplementary Table 1 — All primers used for vector construction and gene expression analysis. [file Table_1.DOCX]

**Table 1 All primers used for vector construction and gene expression analysis.**

| **Primers** | **Sequence** |
| --- | --- |
| *Pn2-ODD*-pRI101-F | GCAAGTTCTTCACTGTTGATAATGACAATCGTCGATGCAAAC |
| *Pn2-ODD*-pRI101-R | GATTCAGAATTCGGATCCCTATGGTTCTAAGGGCTGCTC |
| *Pn2-ODD*-pTFH15.3-F | CAGGCCGGCGCGCCACCCGGGATGACAATCGTCGATGCAA |
| P*n2-ODD-pTFH15.3-R* | AATCGGCCGGGCCCTCCCGGGCTATGGTTCTAAGGGCTGC |
| *Pn2-ODD*-qF | GCAGAGCACCGAGTTGTGACGAA |
| *Pn2-ODD*-qR | TTGCCATTAAGCCCAGTTGAGTA |
| AtTubulin-qF | AAGGGACACTACACGGAAGGAG |
| AtTubulin-qR | TGACCAGGGAACCTCAGACAGC |
| AtFeSOD1-qF | AGTGCTGTCACCGCAAACTACG |
| AtFeSOD1-qR | TATGCGGCTCCAAAGCATCCAG |
| AtFeSOD2-qF | GGTGGCTGTTTCCGGTGTTATC |
| AtFeSOD2-qR | TATGCGGTTCCAGAGCATCAAG |
| AtCAT1-qF | TCGGGAAGGAGAACAACTTCAAGC |
| AtCAT1-qR | TCACGAATCGTTCTTGCCTGTC |
| AtCAT2-qF | AAGTATCCAACTCCGCCTGCTG |
| AtCAT2-qR | TGGATGAATCGTTCTTGCCTCTC |
| AtCAT3-qF | AGGTACAGATCATGGGCACCAG |
| AtCAT3-qR | AAGGATCGATCAGCCTGAGACC |
| AtCu/ZnSOD1-qF | AACGGTTGCATGTCTACTGGTC |
| AtCu/ZnSOD1-qR | GTGATTGTGAAGGTGGCAGTTCC |
| AtCu/ZnSOD2-qF | CAGGGCCTCATGGATTTCATCTCC |
| AtCu/ZnSOD2-qR | TGGAGCTCCGTGTGTCATGTTG |
| AtCu/ZnSOD3-qF | AACAACGTCCGAGGCTGTCTTC |
| AtCu/ZnSOD3-qR | TTCCCGGTGACATGAGTAGTTCCG |
| AtRbohD-qF | ATGATCAAGGTGGCTGTTTACCC |
| AtRbohD-qR | ATCCTTGTGGCTTCGTCATGTG |
| AtHKT1-qF | TCAGTGCATATGGAAACGTTGG |
| AtHKT1-qR | CCATTGGACTCCATCGTCCTG |
| AtSOS3-qF | CGCTTCTTCACGAATCCGAACTAGTTC |
| AtSOS3-qR | GGCAAAGTCATGTTCTTGATGAGCGATG |
| AtP5CS1-qF | GGTTTCCGATTTGGACTTGGTGCAG |
| AtP5CS1-qR | CCATTGTCTCCGTCGACAACTTGTC |
| AtNHX1-qF | AGTGTCGAAACTGCCTTCGT |
| AtNHX1-qR | CCAGTGCCTAGCCCAATCAA |
| AtMYB2-qF | AACGTCTTCGAATTCTCCGGCTGA |
| AtMYB2-qR | ATCGTTGAACTCTCCGAAACCCGT |
| AtAREB1-qF | AGAGTCAGAGTCAGAGTCAGAG |
| AtAREB1-qR | TTACACCAGCGGCAACAAC |
| AtDREB2B-qF | TGCAGATTACGGTTGGCCTT |
| AtDREB2B-qR | CATCGTGTGGCTCGAGATGA |
| PpActin-qF | TGGCGATTCAGGCAGTGTTGTC |
| PpActin-qR | CATCAGTGCGTCCGTCAAGTCG |
| PpSHP1-qF | CACGTTCGTTGATGTGCTGTTG |
| PpSHP1-qR | GCAAGTAACCCAGGATTGTCAG |
| PpSHP2-qF | TTAGCTATTCTGTTGCCGCCTCT |
| PpSHP2-qR | GACGTAAAGCGCATACAAGATCC |
| PpABI3a-qF | ATGCGTCGTTGTCTTCATATTTAAT |
| PpABI3a-qR | GGGTTCATCTGAGGCGGGTTTA |
| PpABI3b-qF | AAAAGTCCCTGCCCATTCGTGAG |
| PpABI3b-qR | ACGTCTACAAAGGTGCCGCTACC |
| PpABI3c-qF | AACGACTGTATGGAGGCGGAC |
| PpABI3c-qR | CAAAGGTTGGAGTGAAGTGGC |
| PpABI5a-qF | TGCTAACGCGATGACGAGCCAG |
| PpABI5a-qR | CCGCTACCCCCACTATCCTCAC |
| PpABI5b-qF | CGAACGCTGAGCATGAAGACG |
| PpABI5b-qR | CCATAAACGAACTGAACCCTTG |
| PpCOR47-qF | AAACGCCCGAGAGCGGTGTT |
| PpCOR47-qR | TGGTGGAGGCTGGAGCAGTGGA |
| PpCORTMC-AP3-qF | AACACCCCCCATGCCGACTTCT |
| PpCORTMC-AP3-qR | CTCCTTGCCCATACGCTGAACG |
