# Supplementary material for: A Moss 2-Oxoglutarate/Fe(II)-Dependent Dioxygenases (2-ODD) Gene of Flavonoids Biosynthesis Positively Regulates Plants Abiotic Stress Tolerance
Source: Front Plant Sci. 2022 Jul 29;13:850062. doi: 10.3389/fpls.2022.850062 (PMC9372559; doi:10.3389/fpls.2022.850062)
Supplement: Supplementary Table 2 — Differential metabolites (36 up-regulate and 12 down-regulate) identified by the flavonoids metabolomics in 5-day-old WT and transgenic Arabidopsis. [file Table_2.DOCX]

**Table 2** **Differential metabolites (36 up-regulate and 12 down-regulate) identified by the flavonoids metabolomics in 5-day-old WT and transgenic *Arabidopsis*.**

| **Index** | **Formula** | **Compounds** | **Class II** | **Type** |
| --- | --- | --- | --- | --- |
| Zmhp003514 | C_16_H_12_O_6_ | 6,7,8-Tetrahydroxy-5-methoxyflavone | Flavonoid | up |
| Lmmn006022 | C_22_H_22_O_11_ | Rhamnetin-3-O-rhamnoside | Flavonoid | up |
| pmb0580 | C_21_H_20_O_9_ | Chrysin-5-O-glucoside (Toringin) | Flavonoid | up |
| HJN087 | C_21_H_22_O_10_ | Naringenin-4'-O-glucoside | Flavonoid | up |
| Zmhp005139 | C_25_H_24_O_15_ | Tamarixetin-3-O-(6''-malonyl)glucoside | Flavonoid | up |
| Zmdp003457 | C_27_H_30_O_17_ | Myricetin-3-O-galactoside-3'-O-rhamnoside | Flavonoid | up |
| Hmjp003248 | C_30_H_30_O_19_ | Luteolin-7-O-(6''-malonyl)glucuronide-5-O-rhamnoside | Flavonoid | up |
| pmb2976 | C_33_H_40_O_20_ | Chrysoeriol-8-C-arabinoside-7-O-Sophoroside | Flavonoid | up |
| Zmjp003179 | C_26_H_28_O_15_ | Orientin-2''-O-xyloside | Flavonoid | up |
| pmb3002 | C_28_H_32_O_15_ | Chrysoeriol-7-O-rutinoside | Flavonoid | up |
| pme0001 | C_28_H_34_O_15_ | Hesperetin-7-O-neohesperidoside(Neohesperidin) | Dihydroflavone | up |
| mws0036 | C_28_H_34_O_15_ | Hesperetin-7-O-rutinoside (Hesperidin) | Dihydroflavone | up |
| mws0057 | C_21_H_22_O_11_ | Eriodictyol-7-O-glucoside | Dihydroflavone | up |
| Lmlp005236 | C_21_H_22_O_11_ | Dihydrokaempferol-3-O-glucoside | Dihydroflavonol | up |
| mws1361 | C_21_H_22_O_11_ | Taxifolin-3-O-rhamnoside (Astilbin) | Dihydroflavonol | up |
| pmb2979 | C_25_H_26_O_14_ | Hesperetin-7-O-(6''-malonyl)glucoside | Dihydroflavonol | up |
| Lmmp004257 | C_22_H_22_O_11_ | 6-C-Methylquercetin-3-O-rhamnoside | Flavonols | up |
| Lmmn003398 | C_23_H_22_O_12_ | Kaempferol-3-O-(6''-acetyl)glucoside | Flavonols | up |
| Lmpp003268 | C_33_H_40_O_20_ | Kaempferol-3-O-rutinoside-7-O-glucoside | Flavonols | up |
| Li512111 | C_24_H_24_O_13_ | Isorhamnetin-3-O-(6''-acetylglucoside) | Flavonols | up |
| Hmln002189 | C_24_H_22_O_15_ | Quercetin-3-O-(6''-malonyl)galactoside | Flavonols | up |
| Hmcp001858 | C_26_H_28_O_14_ | Kaempferol-3-O-arabinoside-7-O-rhamnoside | Flavonols | up |
| Hmcp001769 | C_26_H_28_O_15_ | Quercetin-3-O-rhamnosyl(1→2)arabinoside | Flavonols | up |
| Lmbp002336 | C_27_H_30_O_16_ | Quercetin-3-O-(2''-O-rhamnosyl)galactoside | Flavonols | up |
| Hmgp001888 | C_28_H_32_O_17_ | Patuletin-3-O-rutinoside | Flavonols | up |
| Hmln001836 | C_29_H_32_O_17_ | Kaempferol-3-O-(6''-Acetyl)glucosyl-(1→3)-Galactoside | Flavonols | up |
| Hmcp001947 | C_31_H_34_O_19_ | Isorhamnetin-3-O-(6''-malonylglucoside)-7-O-rhamnoside | Flavonols | up |
| Lmdp004696 | C_38_H_40_O_20_ | Kaempferol-3-O-(6''-Sinapyl)glucosyl-(1→2)-Galactoside | Flavonols | up |
| pmp001312 | C_33_H_40_O_22_ | 6-Hydroxykaempferol-3,7,6-O-triglycoside | Flavonols | up |
| pmb0550 | C_21_H_21_O_11+_ | Cyanidin-3-O-glucoside (Kuromanin) | Anthocyanins | up |
| pme1777 | C_27_H_31_O_16+_ | Cyanidin-3,5-O-diglucoside (Cyanin) | Anthocyanins | up |
| Lmjp001323 | C_32_H_39_O_20+_ | Cyanidin-3-O-(2''-O-xylosyl)glucoside-5-O-glucoside | Anthocyanins | up |
| HJN041 | C_21_H_24_O_11_ | Epicatechin glucoside | Flavanols | up |
| pma0791 | C_24_H_24_O_13_ | Naringenin-7-O-(6''-malonyl)glucoside | Flavanols | up |
| Lmlp006175 | C_21_H_22_O_10_ | Isosalipurposide (Phlorizin Chalcone) | Chalcones | up |
| pmb0676 | C_38_H_40_O_19_ | Chrysoeriol-8-C-glucoside-7-O-(6''-feruloyl)glucoside | Flavonoid carbonoside | up |
| pme0376 | C_15_H_12_O_5_ | Naringenin (5,7,4'-Trihydroxyflavanone) | Dihydroflavone | Down |
| pme3475 | C_15_H_12_O_5_ | Butin | Dihydroflavone | Down |
| pmb0592 | C_34_H_42_O_20_ | Chrysoeriol-7-O-rutinoside-5-O-glucoside | Flavonoid | Down |
| pmb0620 | C_34_H_42_O_21_ | Chrysoeriol-6,8-di-C-glucoside-7-O-glucoside | Flavonoid | Down |
| pmb0578 | C_32_H_30_O_15_ | Luteolin-7-O-(6''-sinapoyl)glucoside | Flavonoid | Down |
| mws0091 | C_21_H_20_O_12_ | Quercetin-3-O-glucoside (Isoquercitrin) | Flavonols | Down |
| mws1329 | C_21_H_20_O_12_ | Quercetin-7-O-glucoside | Flavonols | Down |
| Lmyp004444 | C_22_H_22_O_12_ | Tricin-4'-methylether-3'-O-glucoside | Flavonols | Down |
| pmn001642 | C_23_H_20_O_13_ | Kaempferol-3-O-(2''-O-acetyl)glucuronide | Flavonols | Down |
| Lmqp002349 | C_36_H_42_O_24_ | Kaempferol-3-O-(6''''-malonyl)sophorotrioside | Flavonols | Down |
| mws0914 | C_15_H_12_O_5_ | Pinobanksin | Dihydroflavonol | Down |
| mws1397 | C_22_H_18_O_10_ | Epicatechin gallate | Flavanols | Down |
